# Efficacy of endoscopic therapy for T1b esophageal cancer and construction of prognosis prediction model: a retrospective cohort study

**DOI:** 10.1097/JS9.0000000000000427

**Published:** 2023-05-03

**Authors:** Xiangli Fan, Jing Wang, Lingling Xia, Hu Qiu, Yuanyuan Tian, Yutian Zhangcai, Xixi Luo, Yuelan Gao, Chen Li, Yong Wu, Wensi Zhao, Jiamei Chen, Wei Shi, Jingping Yuan, Shaobo Ke, Yongshun Chen

**Affiliations:** a Cancer Center; b Eye Center; Departments of c Pharmacy; d Pathology, Renmin Hospital of Wuhan University, Wuhan, Hubei Province, P. R. China

**Keywords:** endoscopic therapy, oesophageal neoplasm, esophagectomy, prognosis prediction, survival analysis

## Abstract

The efficacy of endoscopic therapy on the long-term survival outcomes of T1b oesophageal cancer (EC) is unclear, this study was designed to clarify the survival outcomes of endoscopic therapy and to construct a model for predicting the prognosis in T1b EC patients.

**Methods:**

This study was performed using the Surveillance, Epidemiology, and End Results (SEER) database from 2004 to 2017 of patients with T1bN0M0 EC. Cancer-specific survival (CSS) and overall survival (OS) were compared between endoscopic therapy group, esophagectomy group and chemoradiotherapy group, respectively. Stabilized inverse probability treatment weighting was used as the main analysis method. The propensity score matching method and an independent dataset from our hospital were used as sensitivity analysis. The least absolute shrinkage and selection operator regression (Lasso) was employed to sift variables. A prognostic model was then established and was verified in two external validation cohorts.

**Results:**

The unadjusted 5-year CSS was 69.5% (95% CI, 61.5–77.5) for endoscopic therapy, 75.0% (95% CI, 71.5–78.5) for esophagectomy and 42.4% (95% CI, 31.0–53.8) for chemoradiotherapy. After stabilized inverse probability treatment weighting adjustment, CSS and OS were similar in endoscopic therapy and esophagectomy groups (*P*=0.32, *P*=0.83), while the CSS and OS of chemoradiotherapy patients were inferior to endoscopic therapy patients (*P*<0.01, *P*<0.01). Age, histology, grade, tumour size, and treatment were selected to build the prediction model. The area under the curve of receiver operating characteristics of 1, 3, and 5 years in the validation cohort 1 were 0.631, 0.618, 0.638, and 0.733, 0.683, 0.768 in the validation cohort 2. The calibration plots also demonstrated the consistency of predicted and actual values in the two external validation cohorts.

**Conclusion:**

Endoscopic therapy achieved comparable long-term survival outcomes to esophagectomy for T1b EC patients. The prediction model developed performed well in calculating the OS of patients with T1b EC.

## Introduction

Highlights

- This study analyzed the survival outcomes of T1b oesophageal cancer undergoing endoscopy therapy versus esophagectomy or chemoradiotherapy.
- Endoscopic therapy as a minimally invasive surgery for organ preservation manifested comparable survival benefits to esophagectomy and was superior to chemoradiotherapy.
- An online interactive dynamic nomogram was developed and validated based on five variables (age, histology, tumour grade, tumour size, and treatment).

With the maturity and prevalence of endoscopic techniques, more superficial oesophageal malignancies are being diagnosed^1,2^. Traditional esophagectomy for superficial oesophageal cancer (EC) is associated with high mortality and incidence of adverse events^3-6^, as well as a negative impact on the long-term quality of life of surviving patients^7^. Therefore, an effective, minimally invasive, or non-surgical alternative to esophagectomy, would be of great clinical value for the treatment of superficial EC, especially for patients who cannot tolerate or are unwilling to undergo surgery. According to Japanese guidelines, endoscopic resection is an alternative treatment for superficial EC invading the epithelium (T1a-M1) or lamina (T1a-M2) but remains an investigational indication for superficial disease invading the mucosal muscle layer (T1a-M3) or submucosa (T1b) because of the risks of lymph node metastasis^8^.

Some authors recommend esophagectomy for T1b EC^9,10^, some think that endoscopic therapy was not inferior to esophagectomy^11–16^, and definitive chemoradiotherapy achieved a comparable efficacy to that of esophagectomy in patients with T1b EC^17–19^. Endoscopic therapy as an organ preservation therapy could reduce hospital stay, hospitalization costs, operation time, perioperative mortality and surgery-related adverse events compared to esophagectomy^11,20–22^. However, some studies have reported that endoscopic therapy was associated with a higher recurrence rate due to the inability to perform lymph node dissection and a lower R0 resection rate^21^. Whether endoscopic therapy negatively impacts the long-term survival of patients with T1b EC remains controversial. Chemoradiotherapy, as another alternative organ preservation therapy, its high local failure rate and adverse events related to dosing escalation also cannot be ignored^23–25^.

In this context, we evaluated the survival outcomes of patients with T1bN0M0 EC treated with endoscopic therapy, esophagectomy, and chemoradiotherapy using the Surveillance, Epidemiology, and End Results (SEER) database. We hypothesized that for individuals with T1bN0M0 EC, endoscopic therapy could be an effective organ preservation therapy. The external dataset from our hospital was used to validate the final results. Subsequently, we developed a convenient web–based calculator to predict the long-term survival of patients with T1b EC.

## Material and methods

### Study design

This was a retrospective study combining the SEER database with clinical data. First, the changes in the treatment modalities of T1bN0M0 EC from 2004 to 2017 based on the SEER database were analyzed, and then the feasibility of endoscopic therapy for T1b EC was evaluated by comparing the cancer-specific survival (CSS) and overall survival (OS) of endoscopic therapy versus esophagectomy and chemoradiotherapy. Different statistical methods and the external dataset from different sources for sensitivity analysis were used to minimize possible bias. Finally, the data of T1b EC patients in the SEER database from 2004 to 2012 was as the training cohort, the data from 2013 to 2017 was as the validation cohort 1 and the data from our hospital was as the validation cohort 2. A network calculator was developed to predict the prognosis of T1b patients with the OS as the outcome, and the model was verified internally and externally. Our study was performed according to the Helsinki Declaration. In addition, ethical approval for this study was provided by the Institutional Review Board of our hospital. This retrospective study has been reported in line with the STROCSS criteria^26^, Supplemental Digital Content 1, http://links.lww.com/JS9/A428.

### Setting and participants

The data included in this study were from the Incidence-SEER 17 Registries Research Plus Data, which was released in April 2022 based on patient information submitted in November 2021. The study population consisted of patients with a primary T1b EC without regional lymph node metastasis and distant metastasis diagnosed between 2004 and 2017. The histological types included only adenocarcinoma and squamous cell carcinoma, and patients who received endoscopic therapy, esophagectomy, or chemoradiotherapy were included. Patients who received preoperative neoadjuvant therapy and had incomplete follow-up information were excluded. In addition, patients with missing data on stage, therapy and tumour grade were also excluded. The same criteria were used to retrospectively collect the information of patients diagnosed with T1b EC in our hospital from January 2016 to June 2021 to form an external dataset. The survive status was determined by telephone or other means of contact in addition to inquiring about medical history records. The follow-up period ended on 1 February 2023.

### Variables

The primary exposure factor, collected in the SEER registry under the variable “Surg Prim Site” was the receipt of endoscopic therapy, esophagectomy and chemoradiotherapy. Endoscopic therapy was the reference group, and esophagectomy or chemoradiotherapy was the exposure group. Surgical codes for endoscopic therapy in the SEER database are 10–14 and 20–27. Codes 10–14 represent local tumour destruction, and no specimens were sent for pathological examination, including photodynamic therapy, electrocautery, cryotherapy, and laser ablation. Codes 20–27 represent local tumour resection, and specimens were sent for pathological examination. Surgical codes for esophagectomy are 30–80. Patient demographic variables were age at diagnosis, treated in 10-year increments, sex, race, and marital status. Tumour and treatment variables were tumour size (≤ 2 cm, > 2 cm, and unknown), grade (I well differentiated, II moderately differentiated, III poorly differentiated, and IV undifferentiated), histology (adenocarcinoma, ICD-O-3 code: 8140-8145, 8210, 8211, 8255, 8260, 8261, 8263, 8310, 8480, 8481, 8490, 8574; squamous cell carcinoma, ICD-O-3 code: 8050-8052, 8070-8076, 8083-8084), primary site (150 cervical oesophagus, 151 thoracic oesophagus, 152 abdominal oesophagus, 153 upper third of oesophagus, 154 middle third of oesophagus, 155 lower third of oesophagus, 158 overlapping lesions of the oesophagus, 159 oesophagus, NOS), adjuvant therapy (given or not given).

In the survival analysis, the primary outcome was CSS at 5 years, and the secondary outcome was OS at 5 years. CSS is determined by the death attributable to the cancer of interest, whereas OS is determined from all causes of death. Both were determined in the SEER database using cancer registry data and death certificates. The cause of death among patients diagnosed in our hospital was determined by death certificate and follow-up. OS was an outcome when establishing and validating the prognosis prediction model.

### Statistical analysis

Continuous variables were expressed as mean and standard deviation or median and interquartile range. Categorical variables were expressed as frequencies and percentages. Group comparisons for continuous data were analyzed by *t*-test or Mann–Whitney U test and categorical data by χ2 test or Fisher exact test. Kaplan–Meier curves and log-rank tests were used to assess CSS and OS. Comparative analyses were conducted using three methods: unadjusted, stabilized inverse probability treatment weighting (sIPTW) and propensity score matching (PSM). As the main analysis method, sIPTW used a multivariate binomial logistic regression model to generate propensity scores. The PSM method as a sensitivity analysis used the nearest neighbour method (not replaced; the caliper is 0.02) to select matching samples, and baseline demographics and clinical characteristics were matched at a 1:1 ratio. To mitigate the impact of missing survival data in the absence of reliable information on the cause of missing data, “missing” was included at a level of “other” or “unknown” for some variables.

LASSO is a shrinkage and variable selection tool that can reduce the complexity and over fitting of predictive models, resulting in simpler and more accurate models than regular regression^27^. In the part of model establishment and validation, LASSO regression was applied to select variables related to survival outcomes, and Cox regression was used to construct the prediction model. The discrimination of the model was assessed by area under the curve of receiver operating characteristic (AUC) value. Calibration graphs were plotted to assess the distance between model predicted and actual outcomes.

Statistical significance was determined by a prespecified 2-sided alpha of 0.05 and 95% confidence interval (CI). SPSS 26.0 and R-4.2.0 were used for analysis.

## Results

### Demographics, clinical characteristics and changes in treatment methods

Of the 988 included individuals, 194 (19.64%) underwent endoscopic therapy, 699 (70.75%) underwent esophagectomy, and 95 (9.61%) underwent chemoradiotherapy. Most patients were men (81.28%) and white (89.70%), with a median age of 67 years (interquartile range, 60–75). The median postintervention follow-up for the entire cohort was 53 months. Endoscopic therapy patients were older, had smaller tumours, and were more likely to undergo postoperative adjuvant therapy, whereas chemoradiotherapy patients had more cervical oesophageal and upper third oesophageal cancer, as well as more squamous cell carcinoma. According to current treatment guidelines, these differences can be expected. Demographic and clinical characteristics of T1bN0M0 EC patients with different treatments are summarized in Table 1.

**Table 1:** Characteristics of all T1bN0M0 esophageal cancer patients stratified by treatment.

	Overall	Endoscopic therapy	Esophagectomy	Chemoradiotherapy
Characteristic	*n*=988	*n*=194	*n*=699	*n*=95
Age, *n* (%)				
<55 years	110 (11.13)	13 (6.70)	92 (13.16)	5 (5.26)
55–64 years	268 (27.13)	31 (15.98)	215 (30.76)	22 (23.16)
65–74 years	360 (36.44)	61 (31.44)	266 (38.05)	33 (34.74)
75–84 years	223 (22.57)	72 (37.11)	120 (17.17)	31 (32.63)
≥85 years	27 (2.73)	17 (8.76)	6 (0.86)	4 (4.21)
Sex, *n* (%)				
Male	803 (81.28)	148 (76.29)	573 (81.97)	82 (86.32)
Female	185 (18.72)	46 (23.71)	126 (18.03)	13 (13.68)
Race, *n* (%)				
White	880 (89.07)	172 (88.66)	636 (90.99)	72 (75.79)
Black	54 (5.47)	9 (4.64)	28 (4.01)	17 (17.89)
Other	54 (5.47)	13 (6.70)	35 (5.01)	6 (6.32)
Marital status, *n* (%)				
Married	644 (65.18)	111 (57.22)	477 (68.24)	56 (58.95)
Single	129 (13.06)	31 (15.98)	88 (12.59)	10 (10.53)
Other	215 (21.76)	52 (26.80)	134 (19.17)	29 (30.53)
Primary site, *n* (%)				
Cervical oesophagus	11 (1.11)	1 (0.52)	4 (0.57)	6 (6.32)
Thoracic oesophagus	34 (3.44)	6 (3.09)	25 (3.58)	3 (3.16)
Abdominal oesophagus	12 (1.21)	1 (0.52)	11 (1.57)	0
Upper third of oesophagus	34 (3.44)	10 (5.15)	15 (2.15)	9 (9.47)
Middle third of oesophagus	135 (13.66)	32 (16.49)	82 (11.73)	21 (22.11)
Lower third of oesophagus	704 (71.26)	130 (67.01)	526 (75.25)	48 (50.53)
Overlapping lesion of oesophagus	17 (1.72)	3 (1.55)	12 (1.72)	2 (2.11)
OEsophagus, NOS	41 (4.15)	11 (5.67)	24 (3.43)	6 (6.32)
Histology, *n* (%)				
Adenocarcinoma	772 (78.14)	148 (76.29)	571 (81.69)	53 (55.79)
Squamous	216 (21.86)	46 (23.71)	128 (18.31)	42 (44.21)
Tumour grade (%)				
Grade I	130 (13.16)	26 (13.40)	97 (13.88)	7 (7.37)
Grade II	534 (54.05)	116 (59.79)	363 (51.93)	55 (57.89)
Grade III–IV	324 (32.79)	52 (26.80)	239 (34.19)	33 (34.74)
Tumour size, *n* (%)				
≤2 cm	504 (51.01)	117 (60.31)	361 (51.65)	26 (27.37)
>2 cm	322 (32.59)	26 (13.40)	267 (38.20)	29 (30.53)
Unknown	162 (16.40)	51 (26.29)	71 (10.16)	40 (42.11)
Adjuvant therapy, *n* (%)				
Radiotherapy	—	9 (4.64)	5 (0.72)	—
Chemotherapy	—	2 (1.03)	5 (0.72)	—
Chemoradiotherapy	—	29 (14.95)	4 (0.57)	—
No adjuvant therapy	—	154 (79.38)	685 (98.00)	—
Cause of death, *n* (%)				
Alive	460 (46.56)	83 (42.78)	356 (50.93)	21 (22.11)
Died, cancer	300 (30.36)	47 (24.23)	200 (28.61)	53 (55.79)
Died, other cause	228 (23.08)	64 (32.99)	143 (20.46)	21 (22.11)
Survival months				
Median (IQR)	53.00 [27.00, 98.00]	41.50 [27.25, 66.75]	65.00 [33.00, 113.00]	21.00 [9.00, 40.50]

IQR, interquartile range; NOS, not otherwise specified.

**Figure 1:**
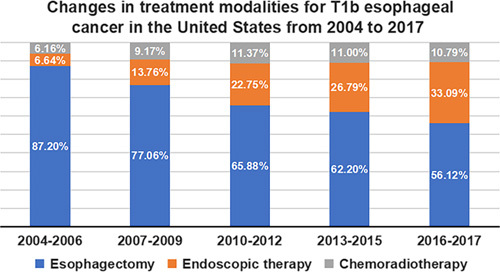
Changes in treatment modalities for T1b oesophageal cancer in the United States from 2004 to 2017.

Endoscopic therapy patients gradually increased from 2004 to 2017 (2004–2006, 6.64%; 2007–2009, 13.76%; 2010–2012, 22.75%; 2013–2015, 26.79%; 2016–2017, 33.09%); esophagectomy patients decreased from 2004 to 2017 (2004–2006, 87.20%; 2007–2009, 77.06%; 2010–2012, 65.88%; 2013–2015, 62.20%; 2016–2017, 56.12%); chemoradiotherapy patients stabilized after 2010 (2004–2006, 6.16%; 2007–2009, 9.17%; 2010–2012, 11.37%; 2013–2015, 11.00%; 2016–2017, 10.79%). Changes in treatment modalities for T1b EC in the United States from 2004 to 2017 are illustrated in Figure 1.

### Survival analysis

#### Survival outcomes before adjustment

An unadjusted survival analysis demonstrated a 5-year CSS of 69.5% (95% CI, 61.5–77.5) for endoscopic therapy, 75.0% (95% CI, 71.5–78.5) for esophagectomy and 42.4% (95% CI, 31.0–53.8) for chemoradiotherapy. The 5-year OS rates were 46.9% (95% CI, 39.1–54.7) for endoscopic therapy, 64.8% (95% CI, 61.1–68.5) for esophagectomy and 26.4% (95% CI, 16.8–36.0) for chemoradiotherapy. It was similar for CSS between endoscopic therapy and esophagectomy groups (*P*=0.58), but the OS of endoscopic therapy patients was inferior to esophagectomy patients (*P*<0.01). The CSS and OS of chemoradiotherapy patients were lower than endoscopic therapy patients (*P*<0.01, *P*<0.01). Kaplan–Meier survival curves for CSS and OS are displayed in Fig. S1, Supplemental Digital Content 2, http://links.lww.com/JS9/A429.

#### Survival outcomes after sIPTW

**Figure 2:**
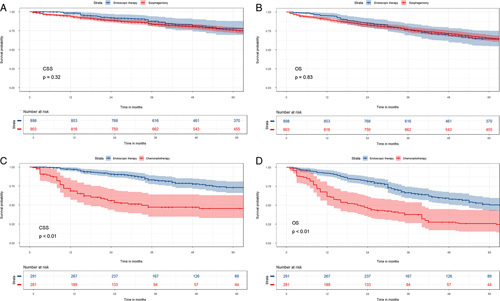
In the sIPTW-adjusted analysis, Kaplan–Meier curves representing CSS (A) or OS (B) in patients with T1b oesophageal cancer who underwent endoscopic therapy and esophagectomy; Kaplan–Meier curves representing CSS (C) or OS (D) in patients with T1b oesophageal cancer who underwent endoscopic therapy and chemoradiotherapy. CSS, cancer-specific survival; sIPTW, stabilized inverse probability of treatment weighting; OS, overall survival.

As the main analysis method, sIPTW can balance intergroup confounding factors without reducing sample size. Before using the sIPTW method to adjust, there were significant differences in age, marital status, primary site, tumour size, and adjuvant therapy between the endoscopic therapy and the esophagectomy groups (Table S1, Supplemental Digital Content 3, http://links.lww.com/JS9/A430); the distributions of race, histology and tumour size were inconsistent between the endoscopic therapy and chemoradiotherapy groups (Table S2, Supplemental Digital Content 3, http://links.lww.com/JS9/A430). After sIPTW adjustment, all covariate differences are balanced (Table S1, Supplemental Digital Content 3, http://links.lww.com/JS9/A430, Table S2, Supplemental Digital Content 3, http://links.lww.com/JS9/A430). The results of survival analysis based on sIPTW adjustment showed that CSS and OS were not significantly different between patients treated with endoscopic therapy and esophagectomy (*P*=0.32, *P*=0.83), but there was a noticeable survival advantage for endoscopic therapy compared with chemoradiotherapy groups (*P*<0.01, *P*<0.01). Kaplan–Meier survival curves for CSS and OS after sIPTW adjustment are displayed in Figure 2.

#### Survival outcomes after PSM

Sensitivity analysis using the PSM method resulted in 136 endoscopic therapy patients matched with 136 esophagectomy patients, and 77 endoscopic therapy patients matched with 77 chemoradiotherapy patients, respectively. After PSM, the baseline characteristics of endoscopic therapy versus esophagectomy and chemoradiotherapy were resembled (Table S3, Supplemental Digital Content 3, http://links.lww.com/JS9/A430 and Table S4, Supplemental Digital Content 3, http://links.lww.com/JS9/A430). Then, survival analysis yielded similar results to those after sIPTW adjustment. Kaplan–Meier survival curves for CSS and OS after PSM adjustment are depicted in Fig. S2, Supplemental Digital Content 2, http://links.lww.com/JS9/A429.

#### Survival outcomes of the external dataset

**Figure 3:**
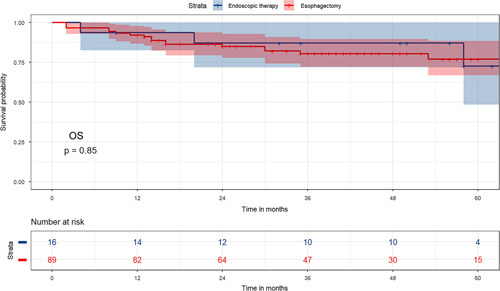
OS comparing endoscopic therapy and esophagectomy groups for T1b oesophageal cancer in the external dataset. OS, overall survival.

To further verify these results, the data of 105 patients diagnosed with T1b EC in our hospital from January 2016 to June 2021, including 16 endoscopic therapy patients and 89 esophagectomy patients, were collected. The majority of the cohort consisted of middle third of oesophagus (80.95%), squamous cell carcinoma (98.10%), Grade II (77.14%), tumour size ≤2 cm (64.76%), and no adjuvant therapy (94.29%). The median age of 63 years and the median follow-up time was 37 months. The baseline characteristics of the external dataset are illustrated in Table S5, Supplemental Digital Content 3, http://links.lww.com/JS9/A430. Due to the retrospective nature of the study, it was difficult to determine the specific cause of death, so only the OS of these patients was analyzed, showing that the OS of endoscopic therapy and esophagectomy patients was similar (*P*=0.85) (Figure. 3).

#### Stratified analysis by histology

Of 216 patients with squamous cell carcinomas, 46 (21.30%) underwent endoscopic therapy, 128 (59.26%) underwent esophagectomy, and 42 (19.44%) underwent chemoradiotherapy. In the sIPTW-adjusted analysis, there was no difference in CSS and OS between endoscopic therapy and esophagectomy groups (*P*=0.63, *P*=0.85), and the CSS and OS of endoscopic therapy group were higher than chemoradiotherapy group (*P*=0.02, *P*=0.03). Of 772 patients with adenocarcinoma, 148 (19.17%) received endoscopic therapy, 571 (73.96%) received esophagectomy, 53 (6.87%) received chemoradiotherapy. In the sIPTW-adjusted analysis for adenocarcinoma, the survival outcomes were in basic agreement with the entire cohort. The comparisons of baseline characteristics stratified by histology are presented in Table S6, Supplemental Digital Content 3, http://links.lww.com/JS9/A430, Table S7, Supplemental Digital Content 3, http://links.lww.com/JS9/A430, Table S8, Supplemental Digital Content 3, http://links.lww.com/JS9/A430 and Table S9, Supplemental Digital Content 3, http://links.lww.com/JS9/A430. Kaplan–Meier survival curves for CSS and OS after sIPTW adjustment are displayed in Fig. S3, Supplemental Digital Content 2, http://links.lww.com/JS9/A429 & S4, Supplemental Digital Content 2, http://links.lww.com/JS9/A429.

### Nomogram construction and validation

#### Construction of the prediction model

The training cohort consisted of 640 EC patients from 2004 to 2012 in the SEER databases, the validation cohort 1 was constitutive of 348 EC patients from 2003 to 2017, and the validation cohort 2 was formed from 105 EC patients in our hospital. The baseline characteristics of training cohort, validation cohort 1 and validation cohort 2 are shown in Table 2.

**Table 2:** Characteristics of training and validation cohorts.

	Training cohort	Validation cohort 1	Validation cohort 2
Characteristic	*n*=640	*n*=348	*n*=105
Age, *n* (%)			
<55 years	77 (12.03)	33 (9.48)	22 (20.95)
55–64 years	185 (28.91)	83 (23.85)	39 (37.14)
65–74 years	226 (35.31)	134 (38.51)	37 (35.24)
75–84 years	132 (20.62)	91 (26.15)	7 (6.67)
≥85 years	20 (3.12)	7 (2.01)	0
Sex, *n* (%)			
Male	543 (84.84)	260 (74.71)	75 (71.43)
Female	97 (15.16)	88 (25.29)	30 (28.57)
Race, *n* (%)			
White	577 (90.16)	303 (87.07)	0
Black	36 (5.62)	18 (5.17)	0
Other	27 (4.22)	27 (7.76)	105 (100.00)
Marital status, *n* (%)			
Married	415 (64.84)	229 (65.80)	101 (96.19)
Single	86 (13.44)	43 (12.36)	4 (3.81)
Other	139 (21.72)	76 (21.84)	0
Primary site, *n* (%)			
Upper third of oesophagus	25 (3.91)	9 (2.59)	6 (5.71)
Middle third of oesophagus	84 (13.12)	51 (14.66)	85 (80.95)
Lower third of oesophagus	457 (71.41)	247 (70.98)	14 (13.33)
Other	74 (11.56)	41 (11.78)	0
Histology, *n* (%)			
Adenocarcinoma	503 (78.59)	269 (77.30)	2 (1.90)
Squamous	137 (21.41)	79 (22.70)	103 (98.10)
Tumour grade, *n* (%)			
Grade I	85 (13.28)	45 (12.93)	11 (10.48)
Grade II	343 (53.59)	191 (54.89)	81 (77.14)
Grade III–IV	212 (33.12)	112 (32.18)	13 (12.38)
Tumour size, *n* (%)			
≤2 cm	297 (46.41)	207 (59.48)	68 (64.76)
>2 cm	226 (35.31)	96 (27.59)	30 (28.57)
Unknown	117 (18.28)	45 (12.93)	7 (6.67)
Treatment, *n* (%)			
Chemoradiotherapy	57 (8.91)	38 (10.92)	0
Endoscopic therapy	92 (14.37)	102 (29.31)	16 (15.24)
Esophagectomy	491 (76.72)	208 (59.77)	89 (84.76)
Survival months			
Median (IQR)	77.00 [27.00, 124.00]	39.00 [27.75, 54.25]	37.00 [23.00, 56.00]

IQR, interquartile range.

**Figure 4:**
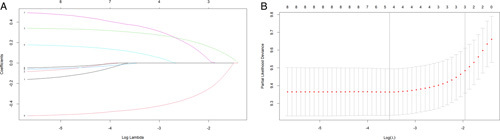
Correlation between coefficient and log lambda (A) and sifting of variables via cross-validation (B) in the LASSO model. LASSO, least absolute shrinkage and selection operator.

**Figure 5:**
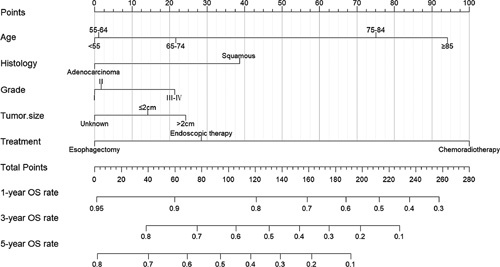
Nomogram displaying the five variables in the prediction model. OS, overall survival.

The significant variables for OS in patients with EC were screened by LASSO regression and cross-validation to obtain two penalty values (λ) (Figure. 4). One is the λ value when the mean square error is minimal, namely λ.min, corresponding to the best precision model, while the other is the λ value obtained within a variance range of the λ.min, namely λ.1se, which corresponds to the model with excellent performance and the least number of independent variables. When λ=λ.1se, we got three variables (age, histology, treatment). After a joint discussion between the Department of Gastroenterology and Cancer center, experts agreed that adding two variables of tumour size and grade was more in line with clinical practice. Therefore, the five variables of age, histology, tumour grade, tumour size, and treatment modality were included. Using the function “cph,” the final prediction model with five variables was constructed to predict the 1-year, 3-year, and 5-year OS (Figure. 5). The older age, squamous cell carcinoma, grade III-IV, tumour size greater than 2 cm, and undergoing chemoradiotherapy were associated with poor survival prognosis.

#### Validation of the prediction model

**Figure 6:**
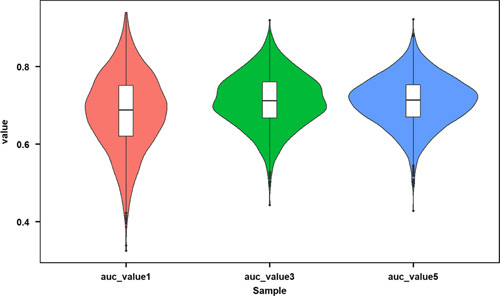
Average AUC values of 1, 3, and 5 years by cross-validation (k=10, times=200). AUC, area under the curve of receiver operating characteristic.

**Figure 7:**
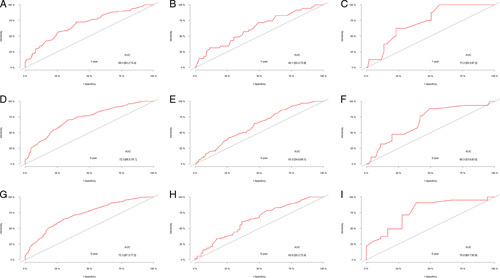
ROC curves of the prediction model for patients with T1b oesophageal cancer. Prediction of 1-year OS in the training group (A), in the validation 1 group (B), and in the validation 2 group (C). Prediction of 3-year OS in the training group (D), in the validation 1 group (E), and in the validation 2 group (F). Prediction of 5-year OS in the training group (G), in the validation 1 group (H), and in the validation 2 group (I). AUC, area under the curve of receiver operating characteristic; OS, overall survival; ROC, receiver operating characteristic.

**Figure 8:**
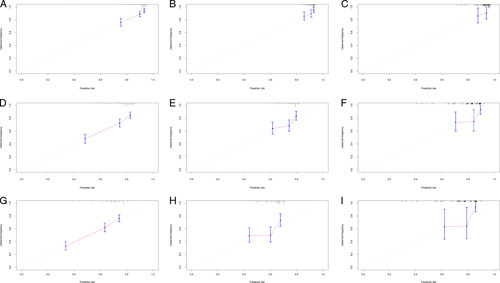
Calibration graphs of the prediction model for patients with T1b oesophageal cancer. 1-year OS in the training group (A), in the validation 1 group (B), and in the validation 2 group (C). 3-year OS in the training group (D), in the validation 1 group (E), and in the validation 2 group (F). 5-year OS in the training group (G), in the validation 1 group (H), and in the validation 2 group (I). OS, overall survival.

For internal validation, cross-validation (k=10, times=200) was performed to prevent over-interpretation of the training cohort data (Figure. 6), showing the average AUC values of 1, 3, and 5 years were 0.683, 0.712, and 0.710. To further evaluate model performance and ensure the external applicability, we validated the model in two external validation cohorts. One cohort was constructed by splitting samples in the SEER database according to calendar time, and the other was composed of patients with T1bN0M0 oesophageal cancer collected in our hospital. The AUC values of 1, 3, and 5 years were 0.693, 0.723, and 0.723 in the training cohort, 0.631, 0.618, and 0.638 in the validation cohort 1, and 0.733, 0.683 and 0.768 in the validation cohort 2, indicating that the prediction model had good discrimination (Figure. 7). The 1-year, 3-year, and 5-year calibration curves of the training and the two validation cohorts did not show large fluctuations, which depicted the predicted results were basically consistent with the actual results in the two cohorts, and the model had good accuracy (Figure. 8).

#### Web‐based calculator

Finally, a dynamic nomogram was constructed using formulas based on prediction models. For T1b EC patients, after inputting values of the five variables into the dynamic nomogram, the assumed time for “Time_death” was provided by ticking the “Predicted Survival at this Follow Up” option, then ticking the “Alpha blending (transparency)” option and clicking “Predict” would show the survival plot, the predicted survival, and the numerical summary of the patient. This dynamic nomogram is available on the website https://predictionmodel1.shinyapps.io/DynNomapp/.

## Discussion

First, the present study demonstrated a decrease in patients with T1b EC who underwent esophagectomy and a gradual increase in endoscopic therapy patients from 2004 to 2017 in the United States. Endoscopic therapy is becoming a trend for the clinical treatment of T1b EC. Secondly, after adjusting for covariate using the sIPTW method, the long-term survival outcomes of endoscopic therapy were comparable to that of esophagectomy and excelled chemoradiotherapy, which supports endoscopic therapy is an effective organ preservation therapy for T1b EC. Sensitivity analysis using the PSM method and independent cohorts from our hospital confirmed these results, which makes our conclusions more reliable.

For the comparison of long-term survival of endoscopic therapy patients and esophagectomy patients, our results are consistent with the results of most of the previous studies^11–16^. However, Otaki *et al.* ^28^. analyzed 73 endoscopic therapy patients and 68 esophagectomy patients with T1b submucosal adenocarcinoma and concluded that esophagectomy was associated with improved overall survival but not cancer-free survival (5-year OS rates, 59% versus 89%, *P*<0.001; 5-year cancer-free survival rates, 69% versus 92%, *P*=0.09). It cannot be ignored that there were large intergroup differences between the endoscopic therapy the esophagectomy groups in this study. More endoscopic therapy patients had high-risk histological factors, whereas the authors did not provide a statistical method to balance the intergroup differences. McCarty *et al.* ^29^. evaluated stage 1 oesophageal cancer in the SEER Database from 2004 to 2015. The stratified analysis included 95 T1b patients with endoscopic therapy and 549 T1b patients with esophagectomy, finding that endoscopic therapy had survival advantages compared with esophagectomy (HR, 3.22, 95% CI, 1.48-7.01; *P*=0.003). The study excluded patients with missing demographic information and unknown tumour size, resulting in too much loss of survival data in the endoscopic therapy group. which may lead to insufficient endoscopic therapy patients reaching study outcome. Compared with previous studies, we had a larger endoscopic therapy cohort and longer follow-up time. Similar results were obtained using two different analysis methods of sIPTW and PSM or evaluating datasets from different sources. Our research seems to be more trustworthy.

JCOG0502, a recent Japanese prospective non-randomized controlled study for T1bN0M0 oesophageal squamous cell carcinoma with 209 esophagectomy patients and 159 chemoradiotherapy patients, demonstrated that chemoradiotherapy was not inferior to esophagectomy (5-year OS rates: 85.5% in chemoradiotherapy versus 86.5% in esophagectomy)^17^. Another study of 156 patients with T1N0M0 oesophageal squamous cell carcinoma (120 in the esophagectomy group and 36 in the chemoradiotherapy group) obtained analogous results^30^. In contrast, our chemoradiotherapy group seems to have a poor survival outcome. Due to the limitations of the SEER database, we lack the performance status and comorbidity information of patients, and we cannot distinguish between radical chemoradiotherapy and palliative chemoradiotherapy. In addition, the follow-up time of our chemoradiotherapy group was limited, most of which did not achieve 5 years. These factors seem to affect the analysis of the survival outcomes of the chemoradiotherapy group. For the efficacy of chemoradiotherapy for T1b EC, large scale, prospective studies are expected.

Patients with high-risk factors for lymph node metastasis, including poor differentiation, lymphovascular invasion, tumour size greater than 2 cm, and invasion depth beyond the superficial one-third into the submucosa^31^, will be more likely to be undertreated with endoscopic resection alone. A Japanese prospective study, JCOG0508, conducted a final analysis of 87 patients with stage I EC who underwent endoscopic resection followed by selective chemoradiotherapy, showing that the 5-year OS of endoscopic resection plus selective chemoradiotherapy was 89.7% comparable to that of esophagectomy^32^. In addition, Lyu *et al.* ^33^. and Naito *et al.* ^34^. reported that radiotherapy or chemoradiotherapy as a supplementary therapy after endoscopic resection could reduce the risk of local, regional or distant metastasis, thereby improving survival outcomes with acceptable toxicities. These studies suggest that for T1b EC patients with a high risk of lymph node metastasis, radiotherapy or chemoradiotherapy as a supplementary therapy after endoscopic resection may achieve a survival outcome not inferior to esophagectomy while preserving organs.

Finally, we selected five variables (age, histology, tumour grade, tumour size, and treatment) related to the OS of T1b EC and constructed a prediction model. By inputting the values of the five variables, we can obtain the 1-year, 3-year, and 5-year OS rates predicted by the model. When evaluating the performance of the prediction model, we performed internal-external validation (using cross-validation for internal validation, and splitting samples in the SEER database by calendar time or using the data from our hospital for external validation), which was considered the first choice in the validation techniques^35^. It is worth mentioning that the validation cohort 1 mainly consisted of patients with oesophageal adenocarcinoma from the United States, while the validation cohort 2 was mostly composed of patients with oesophageal squamous cell carcinoma from China. The prediction model we constructed had been well verified in both cohorts, which strongly demonstrates the generalization of the model. To the best of our knowledge, this is the first study to perform internal-external validation of the survival prediction model with T1b EC. We uploaded the model to a public website to facilitate clinical using. It is worth noting that the prediction model remained constant over time, but the outcome of patients with T1b EC would change as treatment improved. Therefore, the performance of the model would become less accurate over time. Furthermore, whether it could improve the satisfaction of patients and doctors need to be supported by more clinical data.

This study also has some limitations. First, the SEER database used preintervention clinical staging determined by endoscopic appearance, endoscopic ultrasound, or computed tomography and positron emission tomography, which can lead to frequent overstaging and understaging. In addition, the SEER database also lacks some patient clinical and demographic data, including comorbidities that affect the treatment decisions of clinicians and may affect outcomes. Clinicians are more likely to recommend endoscopic therapy or chemoradiotherapy for patients with increased comorbidities. Submucosal invasion depth and lymphovascular invasion are associated with the risk of lymph node metastasis^36^, which affect the long-term survival of T1b oesophageal cancer, and socioeconomic status is associated with oesophageal cancer survival^37^. None of this information is available in the SEER database. Furthermore, the specific regimens for patients undergoing chemotherapy and the doses for patients undergoing radiotherapy are not provided in the database. Finally, an important limitation is that we cannot assess tumour recurrence after treatment, and endoscopic therapy may be associated with an increased risk of recurrence^38^.

## Conclusion

Herein, we observed an upward trend in endoscopic therapy in recent years by analyzing the T1b EC in the SEER database from 2004 to 2017, and endoscopic therapy had a long-term survival outcome comparable to esophagectomy and superior to chemoradiotherapy. Moreover, we developed a well-performing network calculator for predicting the OS rate of T1b EC.

## Ethical approval

Ethical approval for this study (Registration No. WDRM2022-K120) was provided by the Ethical Committee of Renmin Hospital of Wuhan University on 11 July 2022.

## Consent

This study did not involve personal privacy and commercial interests and collected patient diagnosis and treatment information retrospectively from the medical system of our hospital, which was almost no risk to patients. After reviewed by the Ethical Committee of Renmin Hospital of Wuhan University, our study was approved exemption from signing informed consent.

## Source of funding

This work was supported by Central Leading Local Science and Technology Development Special Foundation (ZYYD2020000169). No benefits in any form have been or will be received from a commercial party related to the subject of this work.

## Author contribution

X.F.: conceptualization, writing—original draft, data curation and formal analysis, prepared all the figures and tables, drafted the manuscript. J.W.: writing—original draft, data curation and formal analysis, prepared all the figures and tables, drafted the manuscript. L.X., H.Q.,Y.T., Y.Z., X.L., Y.G., C.L., Y.L.: data curation and formal analysis, prepared all the figures and tables. W.Z., J.C., W.S., J.Y.: validation and drafted the manuscript. S.K., Y.C.: conceptualization, supervision, writing—review and editing. All the authors reviewed and approved the final manuscript.

## Conflicts of interest disclosure

All authors declare no conflicts of interest.

## Research Registration Unique Identifying Number (UIN)

1. Name of the registry: Efficacy of endoscopic therapy for T1b oesophageal cancer and construction of prognosis prediction model: A retrospective cohort study
2. Unique Identifying number or registration ID: researchregistry8811
3. Hyperlink to your specific registration (must be publicly accessible and will be checked): https://www.researchregistry.com/browse-theregistry#home/registrationdetails/642baf1075f889002989583b/.

## Guarantor

Yongshun Chen, MD, PhD, Cancer Center, Renmin Hospital of Wuhan University, Wuhan 430060, Hubei Province, P. R. China”.

## Data availability statement

The datasets used and analyzed during the current study are available from the corresponding author on reasonable request.

## Provenance and peer review

Not commissioned, externally peer-reviewed.

## Supplementary Material
