# Supplementary material for: Efficacy of endoscopic therapy for T1b esophageal cancer and construction of prognosis prediction model: a retrospective cohort study
Source: Int J Surg. 2023 May 3;109(6):1708–19. doi: 10.1097/JS9.0000000000000427 (PMC10389357; doi:10.1097/JS9.0000000000000427)
Supplement: Supplementary file 2 [file js9-109-1708-s002.docx]

**Supplementary figures**

**
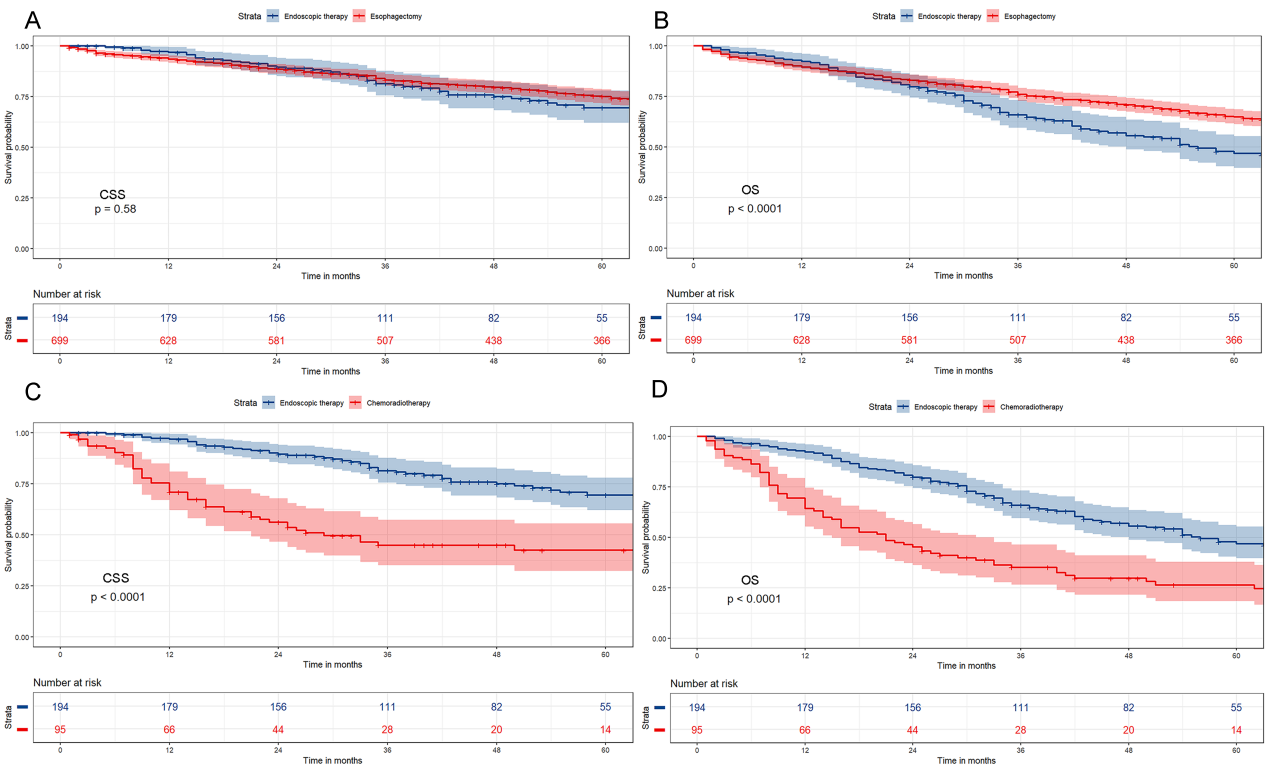
Fig. S1.** Unadjusted Kaplan-Meier curves for CSS (A) or OS (B) comparing endoscopic therapy and esophagectomy groups for T1b esophageal cancer. Unadjusted Kaplan-Meier curves for CSS (C) or OS (D) comparing endoscopic therapy and chemoradiotherapy groups for T1b esophageal cancer. *CSS*, cancer-specific survival; *OS*, overall survival.


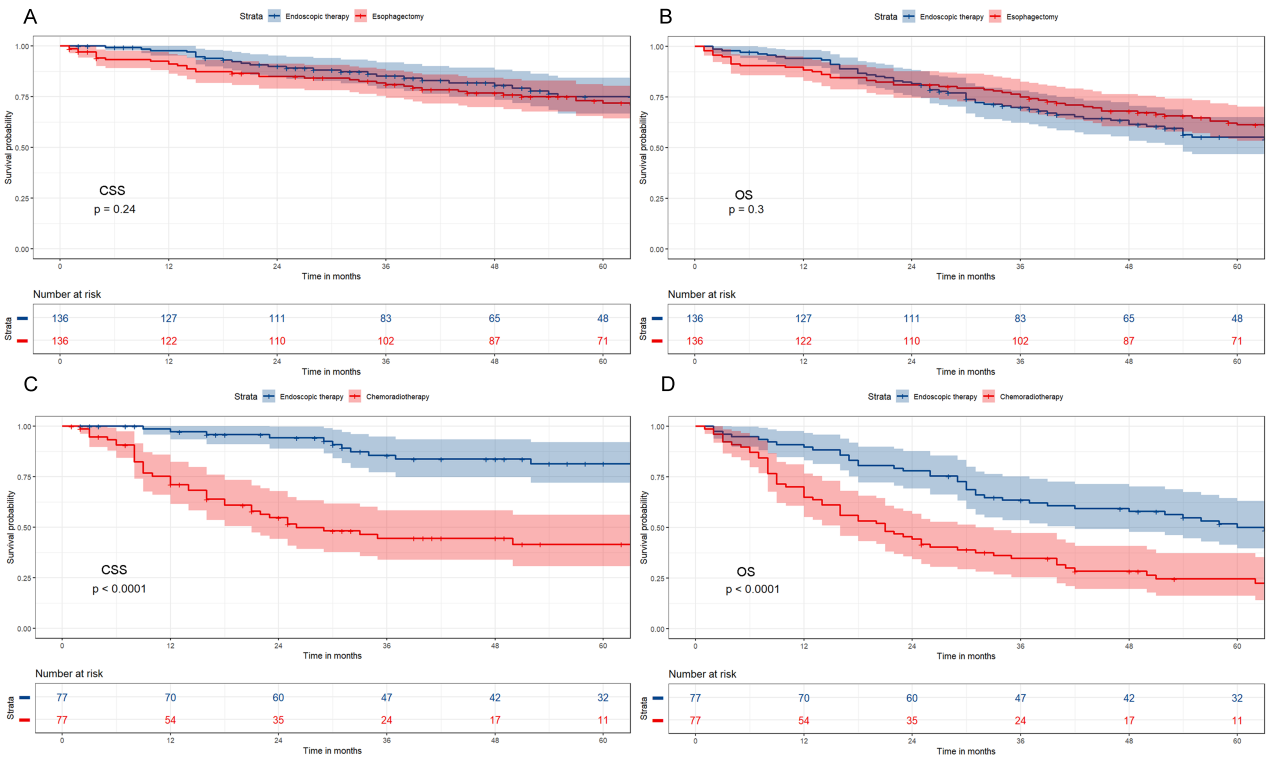
**Fig. S2.** In the PSM-adjusted analysis, Kaplan-Meier curves displaying CSS (A) and OS (B) in subjects for T1b esophageal cancer treated with endoscopic therapy or esophagectomy; Kaplan-Meier curves displaying CSS (C) and OS (D) in subjects for T1b esophageal cancer treated with endoscopic therapy or chemoradiotherapy. *PSM*, propensity score matching; *CSS*, cancer-specific survival; *OS*, overall survival.


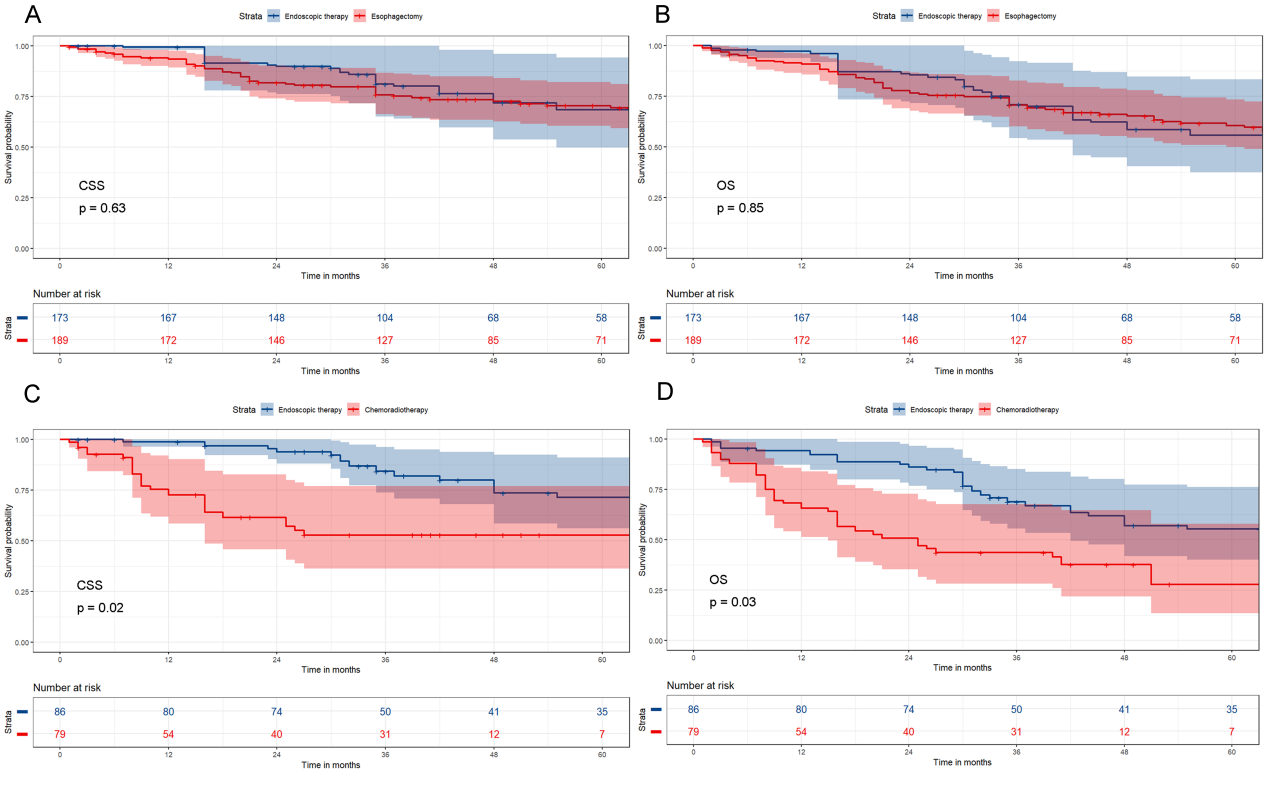
**Fig. S3.** In the sIPTW-adjusted analysis, Kaplan-Meier curves for CSS (A) or OS (B) in patients with T1b esophageal squamous cell carcinoma who received endoscopic therapy and esophagectomy; Kaplan-Meier curves for CSS (C) or OS (D) in patients with T1b esophageal squamous cell carcinoma who received endoscopic therapy and chemoradiotherapy. *sIPTW*, stabilized inverse probability of treatment weighting; *CSS*, cancer-specific survival; *OS*, overall survival.


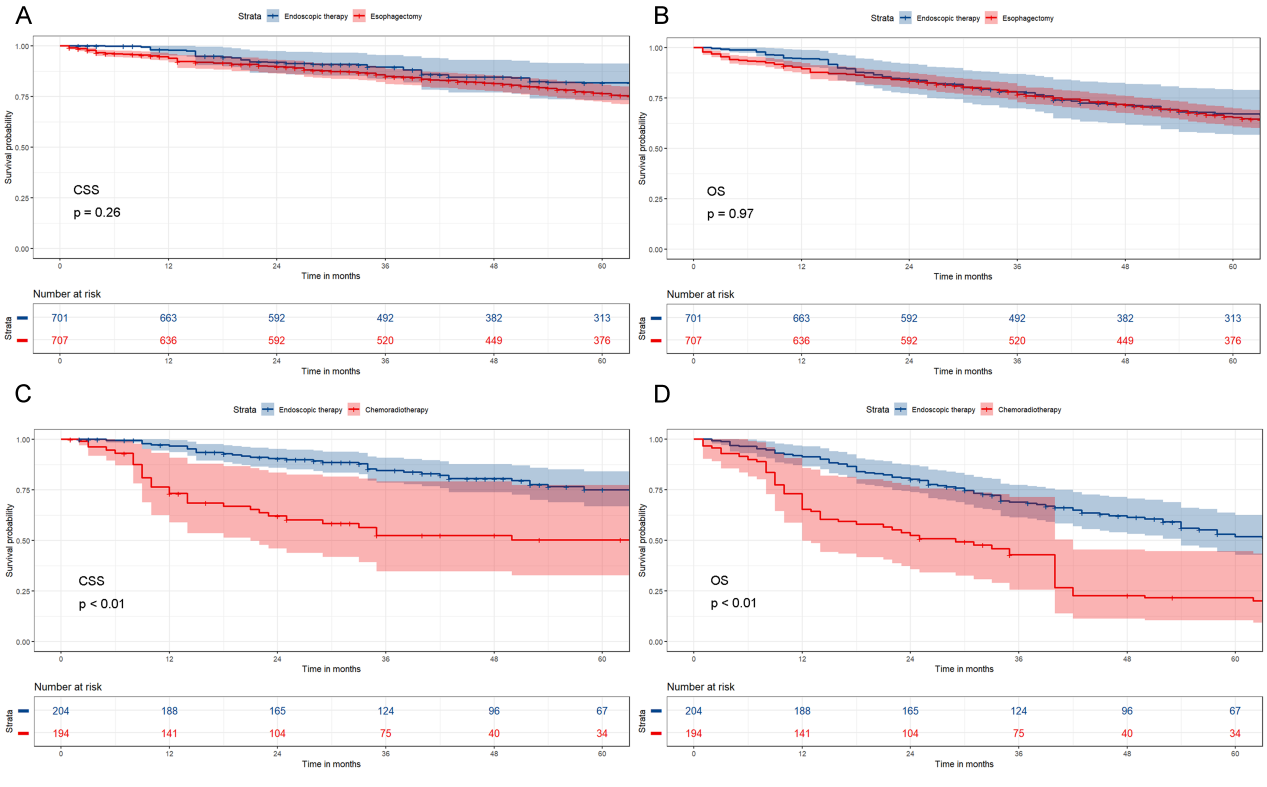
**Fig. S4.** In the sIPTW-adjusted analysis, Kaplan-Meier curves for CSS (A) or OS (B) in patients with T1b esophageal adenocarcinoma who received endoscopic therapy and esophagectomy; Kaplan-Meier curves for CSS (C) or OS (D) in patients with T1b esophageal adenocarcinoma who received endoscopic therapy and chemoradiotherapy. *sIPTW*, stabilized inverse probability of treatment weighting; *CSS*, cancer-specific survival; *OS*, overall survival.
