# Supplementary material for: Efficacy of endoscopic therapy for T1b esophageal cancer and construction of prognosis prediction model: a retrospective cohort study
Source: Int J Surg. 2023 May 3;109(6):1708–19. doi: 10.1097/JS9.0000000000000427 (PMC10389357; doi:10.1097/JS9.0000000000000427)
Supplement: Supplementary file 3 [file js9-109-1708-s003.docx]

**Supplementary tables**

| **Table S1. Baseline characteristics of endoscopic therapy and esophagectomy groups before and after sIPTW** | | | | | | |
| --- | --- | --- | --- | --- | --- | --- |
|  | Unmatched | |  | After sIPTW | |  |
| Group | Endoscopic therapy | Esophagectomy | *P* | Endoscopic therapy | Esophagectomy | *P* |
| N | 194 | 699 |  | 897.5 | 902.7 |  |
| Age (%) |  |  | <0.001 |  |  | 0.960 |
| <55 y | 13 (6.7) | 92 (13.2) |  | 98.7 (11.0) | 104.8 (11.6) |  |
| 55-64 y | 31 (16.0) | 215 (30.8) |  | 233.8 (26.0) | 242.8 (26.9) |  |
| 65-74 y | 61 (31.4) | 266 (38.1) |  | 350.7 (39.1) | 325.2 (36.0) |  |
| 75-84 y | 72 (37.1) | 120 (17.2) |  | 191.0 (21.3) | 212.3 (23.5) |  |
| ≥85 y | 17 (8.8) | 6 (0.9) |  | 23.3 (2.6) | 17.6 (1.9) |  |
| Sex (%) |  |  | 0.094 |  |  | 0.250 |
| Male | 148 (76.3) | 573 (82.0) |  | 748.9 (83.4) | 703.4 (77.9) |  |
| Female | 46 (23.7) | 126 (18.0) |  | 148.7 (16.6) | 199.3 (22.1) |  |
| Race (%) |  |  | 0.592 |  |  | 0.335 |
| White | 172 (88.7) | 636 (91.0) |  | 793.5 (88.4) | 820.6 (90.9) |  |
| Black | 9 (4.6) | 28 (4.0) |  | 24.0 (2.7) | 35.8 (4.0) |  |
| Other | 13 (6.7) | 35 (5.0) |  | 80.0 (8.9) | 46.3 (5.1) |  |
| Marital status (%) |  |  | 0.015 |  |  | 0.460 |
| Married | 111 (57.2) | 477 (68.2) |  | 577.6 (64.4) | 582.1 (64.5) |  |
| Single | 31 (16.0) | 88 (12.6) |  | 100.9 (11.2) | 141.8 (15.7) |  |
| Other | 52 (26.8) | 134 (19.2) |  | 219.0 (24.4) | 178.9 (19.8) |  |
| Primary site (%) |  |  | 0.029 |  |  | 0.800 |
| Upper third of esophagus | 10 (5.2) | 15 (2.1) |  | 20.9 (2.3) | 22.5 (2.5) |  |
| Middle third of esophagus | 32 (16.5) | 82 (11.7) |  | 129.1 (14.4) | 132.7 (14.7) |  |
| Lower third of esophagus | 130 (67.0) | 526 (75.3) |  | 675.0 (75.2) | 651.8 (72.2) |  |
| Other | 22 (11.3) | 76 (10.9) |  | 72.6 (8.1) | 95.6 (10.6) |  |
| Histology (%) |  |  | 0.115 |  |  | 0.861 |
| Adenocarcinoma | 148 (76.3) | 571 (81.7) |  | 688.8 (76.7) | 702.6 (77.8) |  |
| Squamous | 46 (23.7) | 128 (18.3) |  | 208.7 (23.3) | 200.1 (22.2) |  |
| Tumor grade (%) |  |  | 0.115 |  |  | 0.947 |
| Grade I | 26 (13.4) | 97 (13.9) |  | 108.6 (12.1) | 119.3 (13.2) |  |
| Grade II | 116 (59.8) | 363 (51.9) |  | 497.0 (55.4) | 499.5 (55.3) |  |
| Grade III-Ⅳ | 52 (26.8) | 239 (34.2) |  | 291.8 (32.5) | 283.9 (31.4) |  |
| Tumor size (%) |  |  | <0.001 |  |  | 0.923 |
| ≤2 cm | 117 (60.3) | 361 (51.6) |  | 499.2 (55.6) | 495.4 (54.9) |  |
| >2 cm | 26 (13.4) | 267 (38.2) |  | 277.1 (30.9) | 292.5 (32.4) |  |
| Unknown | 51 (26.3) | 71 (10.2) |  | 121.2 (13.5) | 114.8 (12.7) |  |
| Adjuvant therapy (%) |  |  | <0.001 |  |  | 0.609 |
| Yes | 40 (20.6) | 14 (2.0) |  | 52.3 (5.8) | 68.4 (7.6) |  |
| No | 154 (79.4) | 685 (98.0) |  | 845.3 (94.2) | 834.4 (92.4) |  |

Abbreviations: sIPTW, Stabilized inverse probability treatment weighting

| **Table S2. Baseline characteristics of endoscopic therapy and chemoradiotherapy groups before and after sIPTW** | | | | | | |
| --- | --- | --- | --- | --- | --- | --- |
|  | Unmatched | |  | After sIPTW | |  |
| Group | Endoscopic therapy | Chemoradiotherapy | *P* | Endoscopic therapy | Chemoradiotherapy | *P* |
| N | 194 | 95 |  | 291.5 | 280.6 |  |
| Age (%) |  |  | 0.355 |  |  | 0.962 |
| <55 y | 13 (6.7) | 5 (5.3) |  | 17.2 (5.9) | 11.4 (4.1) |  |
| 55-64 y | 31 (16.0) | 22 (23.2) |  | 47.8 (16.4) | 43.5 (15.5) |  |
| 65-74 y | 61 (31.4) | 33 (34.7) |  | 93.4 (32.1) | 87.8 (31.3) |  |
| 75-84 y | 72 (37.1) | 31 (32.6) |  | 112.0 (38.4) | 119.2 (42.5) |  |
| ≥85 y | 17 (8.8) | 4 (4.2) |  | 21.0 (7.2) | 18.7 (6.7) |  |
| Sex (%) |  |  | 0.067 |  |  | 0.561 |
| Male | 148 (76.3) | 82 (86.3) |  | 229.6 (78.8) | 208.8 (74.4) |  |
| Female | 46 (23.7) | 13 (13.7) |  | 61.9 (21.2) | 71.8 (25.6) |  |
| Race (%) |  |  | 0.001 |  |  | 0.781 |
| White | 172 (88.7) | 72 (75.8) |  | 244.0 (83.7) | 242.0 (86.3) |  |
| Black | 9 (4.6) | 17 (17.9) |  | 28.8 (9.9) | 26.2 (9.3) |  |
| Other | 13 (6.7) | 6 (6.3) |  | 18.6 (6.4) | 12.4 (4.4) |  |
| Marital status (%) |  |  | 0.431 |  |  | 0.259 |
| Married | 111 (57.2) | 56 (58.9) |  | 169.4 (58.1) | 189.4 (67.5) |  |
| Single | 31 (16.0) | 10 (10.5) |  | 37.4 (12.8) | 20.9 (7.5) |  |
| Other | 52 (26.8) | 29 (30.5) |  | 84.7 (29.1) | 70.2 (25.0) |  |
| Primary site (%) |  |  | 0.052 |  |  | 0.958 |
| Upper third of esophagus | 10 (5.2) | 9 (9.5) |  | 20.8 (7.1) | 25.4 (9.0) |  |
| Middle third of esophagus | 32 (16.5) | 21 (22.1) |  | 56.4 (19.4) | 55.1 (19.6) |  |
| Lower third of esophagus | 130 (67.0) | 48 (50.5) |  | 179.2 (61.5) | 164.2 (58.5) |  |
| Other | 22 (11.3) | 17 (17.9) |  | 35.1 (12.0) | 35.9 (12.8) |  |
| Histology (%) |  |  | 0.001 |  |  | 0.829 |
| Adenocarcinoma | 148 (76.3) | 53 (55.8) |  | 200.4 (68.7) | 188.6 (67.2) |  |
| Squamous | 46 (23.7) | 42 (44.2) |  | 91.1 (31.3) | 91.9 (32.8) |  |
| Tumor grade (%) |  |  | 0.179 |  |  | 0.833 |
| Grade I | 26 (13.4) | 7 ( 7.4) |  | 31.9 (10.9) | 32.7 (11.7) |  |
| Grade II | 116 (59.8) | 55 (57.9) |  | 174.1 (59.7) | 154.4 (55.0) |  |
| Grade III-Ⅳ | 52 (26.8) | 33 (34.7) |  | 85.5 (29.3) | 93.5 (33.3) |  |
| Tumor size (%) |  |  | <0.001 |  |  | 0.995 |
| ≤2 cm | 117 (60.3) | 26 (27.4) |  | 142.9 (49.0) | 137.2 (48.9) |  |
| >2 cm | 26 (13.4) | 29 (30.5) |  | 63.5 (21.8) | 60.1 (21.4) |  |
| Unknown | 51 (26.3) | 40 (42.1) |  | 85.0 (29.2) | 83.3 (29.7) |  |

Abbreviations: sIPTW, Stabilized inverse probability treatment weighting

| **Table S3. Baseline characteristics of endoscopic therapy and esophagectomy groups before and after PSM** | | | | | | |
| --- | --- | --- | --- | --- | --- | --- |
|  | Unmatched | | | After PSM | | |
| Group | Endoscopic therapy | Esophagectomy | *P* | Endoscopic therapy | Esophagectomy | *P* |
| N | 194 | 699 |  | 136 | 136 |  |
| Age (%) |  |  | <0.001 |  |  | 0.938 |
| <55 y | 13 (6.7) | 92 (13.2) |  | 12 (8.8) | 14 (10.3) |  |
| 55-64 y | 31 (16.0) | 215 (30.8) |  | 20 (14.7) | 17 (12.5) |  |
| 65-74 y | 61 (31.4) | 266 (38.1) |  | 49 (36.0) | 49 (36.0) |  |
| 75-84 y | 72 (37.1) | 120 (17.2) |  | 51 (37.5) | 50 (36.8) |  |
| ≥85 y | 17 (8.8) | 6 (0.9) |  | 4 (2.9) | 6 (4.4) |  |
| Marital status (%) |  |  | 0.015 |  |  | 0.816 |
| Married | 111 (57.2) | 477 (68.2) |  | 85 (62.5) | 80 (58.8) |  |
| Single | 31 (16.0) | 88 (12.6) |  | 18 (13.2) | 19 (14.0) |  |
| Other | 52 (26.8) | 134 (19.2) |  | 33 (24.3) | 37 (27.2) |  |
| Primary site (%) |  |  | 0.029 |  |  | 0.331 |
| Upper third of esophagus | 10 (5.2) | 15 (2.1) |  | 5 (3.7) | 4 (2.9) |  |
| Middle third of esophagus | 32 (16.5) | 82 (11.7) |  | 15 (11.0) | 12 ( 8.8) |  |
| Lower third of esophagus | 130 (67.0) | 526 (75.3) |  | 98 (72.1) | 110 (80.9) |  |
| Other | 22 (11.3) | 76 (10.9) |  | 18 (13.2) | 10 (7.4) |  |
| Tumor size (%) |  |  | <0.001 |  |  | 0.933 |
| ≤2 cm | 117 (60.3) | 361 (51.6) |  | 81 (59.6) | 78 (57.4) |  |
| >2 cm | 26 (13.4) | 267 (38.2) |  | 24 (17.6) | 25 (18.4) |  |
| Unknown | 51 (26.3) | 71 (10.2) |  | 31 (22.8) | 33 (24.3) |  |
| Adjuvant therapy (%) |  |  | <0.001 |  |  | 1.000 |
| Yes | 40 (20.6) | 14 (2.0) |  | 8 (5.9) | 7 (5.1) |  |
| No | 154 (79.4) | 685 (98.0) |  | 128 (94.1) | 129 (94.9) |  |

Abbreviations: PSM, propensity score matching

| **Table S4. Baseline characteristics of endoscopic therapy and chemoradiotherapy groups before and after PSM** | | | | | | |
| --- | --- | --- | --- | --- | --- | --- |
|  | Unmatched | |  | After PSM | |  |
| Group | Endoscopic therapy | Chemoradiotherapy | *P* | Endoscopic therapy | Chemoradiotherapy | *P* |
| N | 194 | 95 |  | 77 | 77 |  |
| Race (%) |  |  | 0.001 |  |  | 1.000 |
| White | 172 (88.7) | 72 (75.8) |  | 68 (88.3) | 68 (88.3) |  |
| Black | 9 (4.6) | 17 (17.9) |  | 8 (10.4) | 8 (10.4) |  |
| Other | 13 (6.7) | 6 (6.3) |  | 1 (1.3) | 1 (1.3) |  |
| Histology (%) |  |  | 0.001 |  |  | 1.000 |
| Adenocarcinoma | 148 (76.3) | 53 (55.8) |  | 49 (63.6) | 49 (63.6) |  |
| Squamous | 46 (23.7) | 42 (44.2) |  | 28 (36.4) | 28 (36.4) |  |
| Tumor size (%) |  |  | <0.001 |  |  | 1.000 |
| ≤2 cm | 117 (60.3) | 26 (27.4) |  | 26 (33.8) | 26 (33.8) |  |
| >2 cm | 26 (13.4) | 29 (30.5) |  | 23 (29.9) | 23 (29.9) |  |
| Unknown | 51 (26.3) | 40 (42.1) |  | 28 (36.4) | 28 (36.4) |  |

Abbreviations: PSM, propensity score matching

| **Table S5. Characteristics of endoscopic therapy and esophagectomy groups in the external data set** | | | | |
| --- | --- | --- | --- | --- |
| Characteristic | Overall | Endoscopic therapy | Esophagectomy | *P* |
|  | n = 105 | n = 16 | n = 89 |  |
| Age (%) |  |  |  | 0.420 |
| <55 y | 22 (20.95) | 1 (6.25) | 21 (23.60) |  |
| 55-64 y | 39 (37.14) | 8 (50.00) | 31 (34.83) |  |
| 65-74 y | 37 (35.24) | 6 (37.50) | 31 (34.83) |  |
| 75-84 y | 7 (6.67) | 1 (6.25) | 6 (6.74) |  |
| Sex (%) |  |  |  | 0.577 |
| Male | 75 (71.43) | 10 (62.50) | 65 (73.03) |  |
| Female | 30 (28.57) | 6 (37.50) | 24 (26.97) |  |
| Marital status (%) |  |  |  | 0.877 |
| Married | 101 (96.19) | 16 (100.00) | 85 (95.51) |  |
| Single | 4 (3.81) | 0 (0.00) | 4 (4.49) |  |
| Primary site (%) |  |  |  | 0.990 |
| Upper third of esophagus | 6 (5.71) | 1 (6.25) | 5 (5.62) |  |
| Middle third of esophagus | 85 (80.95) | 13 (81.25) | 72 (80.90) |  |
| Lower third of esophagus | 14 (13.33) | 2 (12.50) | 12 (13.48) |  |
| Histology (%) |  |  |  | 0.698 |
| Adenocarcinoma | 2 (1.90) | 1 (6.25) | 1 (1.12) |  |
| Squamous | 103 (98.10) | 15 (93.75) | 88 (98.88) |  |
| Tumor grade (%) |  |  |  | 0.495 |
| Grade I | 11 (10.48) | 3 (18.75) | 8 (8.99) |  |
| Grade II | 81 (77.14) | 11 (68.75) | 70 (78.65) |  |
| Grade III | 13 (12.38) | 2 (12.50) | 11 (12.36) |  |
| Tumor size (%) |  |  |  | 0.090 |
| ≤2 cm | 68 (64.76) | 8 (50.00) | 60 (67.42) |  |
| >2 cm | 30 (28.57) | 5 (31.25) | 25 (28.09) |  |
| Unknown | 7 (6.67) | 3 (18.75) | 4 (4.49) |  |
| Adjuvant therapy (%) |  |  |  | 0.226 |
| Yes | 6 (5.71) | 2 (12.50) | 4 (4.49) |  |
| No | 99(94.29) | 14 (87.50) | 85 (95.51) |  |
| Status(%) |  |  |  | 1.000 |
| Alive | 85 (80.95) | 13 (81.25) | 72 (80.90) |  |
| Dead | 20 (19.05) | 3 (18.75) | 17 (19.10) |  |
| Survival months |  |  |  | 0.210 |
| Median (IQR) | 37.00 [23.00, 56.00] | 49.50 [29.50, 59.00] | 37.00 [23.00, 53.00] |  |

Abbreviations: IQR, interquartile range

| **Table S6. Baseline characteristics of endoscopic therapy and esophagectomy groups in ESCC before and after sIPTW** | | | | | | |
| --- | --- | --- | --- | --- | --- | --- |
|  | Unmatched | |  | After sIPTW | |  |
| Group | Endoscopic therapy | Esophagectomy | *P* | Endoscopic therapy | Esophagectomy | *P* |
| N | 46 | 128 |  | 172.9 | 189.2 |  |
| Age (%) |  |  | 0.148 |  |  | 0.695 |
| <55 y | 4 (8.7) | 16 (12.5) |  | 7.9 (4.6) | 18.2 (9.6) |  |
| 55-64 y | 11 (23.9) | 33 (25.8) |  | 37.5 (21.7) | 39.0 (20.6) |  |
| 65-74 y | 13 (28.3) | 51 (39.8) |  | 77.8 (45.0) | 63.4 (33.5) |  |
| 75-84 y | 15 (32.6) | 26 (20.3) |  | 45.3 (26.2) | 65.2 (34.5) |  |
| ≥85 y | 3 (6.5) | 2 (1.6) |  | 4.4 (2.5) | 3.4 (1.8) |  |
| Sex (%) |  |  | 0.544 |  |  | 0.303 |
| Male | 21 (45.7) | 67 (52.3) |  | 103.0 (59.6) | 86.6 (45.8) |  |
| Female | 25 (54.3) | 61 (47.7) |  | 69.9 (40.4) | 102.6 (54.2) |  |
| Race (%) |  |  | 0.328 |  |  | 0.459 |
| White | 28 (60.9) | 93 (72.7) |  | 114.4 (66.2) | 140.9 (74.5) |  |
| Black | 9 (19.6) | 17 (13.3) |  | 17.9 (10.4) | 23.0 (12.1) |  |
| Other | 9 (19.6) | 18 (14.1) |  | 40.6 (23.5) | 25.3 (13.4) |  |
| Marital status (%) |  |  | 0.165 |  |  | 0.499 |
| Married | 22 (47.8) | 77 (60.2) |  | 111.6 (64.5) | 96.5 (51.0) |  |
| Single | 7 (15.2) | 22 (17.2) |  | 29.2 (16.9) | 51.7 (27.3) |  |
| Other | 17 (37.0) | 29 (22.7) |  | 32.2 (18.6) | 41.0 (21.7) |  |
| Primary site (%) |  |  | 0.212 |  |  | 0.995 |
| Upper third of esophagus | 8 (17.4) | 11 (8.6) |  | 13.8 (8.0) | 15.2 (8.0) |  |
| Middle third of esophagus | 22 (47.8) | 55 (43.0) |  | 86.7 (50.1) | 94.9 (50.2) |  |
| Lower third of esophagus | 10 (21.7) | 45 (35.2) |  | 51.8 (30.0) | 54.4 (28.8) |  |
| Other | 6 (13.0) | 17 (13.3) |  | 20.6 (11.9) | 24.6 (13.0) |  |
| Tumor grade (%) |  |  | 0.313 |  |  | 0.534 |
| Grade I | 3 (6.5) | 16 (12.5) |  | 6.6 (3.8) | 18.3 (9.7) |  |
| Grade II | 31 (67.4) | 71 (55.5) |  | 111.0 (64.2) | 120.6 (63.7) |  |
| Grade III-Ⅳ | 12 (26.1) | 41 (32.0) |  | 55.4 (32.0) | 50.3 (26.6) |  |
| Tumor size (%) |  |  | <0.001 |  |  | 0.453 |
| ≤2 cm | 25 (54.3) | 59 (46.1) |  | 67.0 (38.8) | 99.9 (52.8) |  |
| >2 cm | 9 (19.6) | 60 (46.9) |  | 84.0 (48.6) | 68.7 (36.3) |  |
| Unknown | 12 (26.1) | 9 (7.0) |  | 21.9 (12.7) | 20.6 (10.9) |  |
| Adjuvant therapy (%) |  |  | <0.001 |  |  | 0.390 |
| Yes | 10 (21.7) | 2 (1.6) |  | 12.7 (7.3) | 29.5 (15.6) |  |
| No | 36 (78.3) | 126 (98.4) |  | 160.2 (92.7) | 159.6 (84.4) |  |

Abbreviations: ESCC, esophageal squamous cell carcinoma; sIPTW, Stabilized inverse probability treatment weighting

| **Table S7. Baseline characteristics of endoscopic therapy and chemoradiotherapy groups in ESCC before and after sIPTW** | | | | | | |
| --- | --- | --- | --- | --- | --- | --- |
|  | Unmatched | |  | After sIPTW | |  |
| Group | Endoscopic therapy | Chemoradiotherapy | *P* | Endoscopic therapy | Chemoradiotherapy | *P* |
| N | 46 | 42 |  | 86 | 78.7 |  |
| Age (%) |  |  | 0.275 |  |  | 0.653 |
| <55 y | 4 (8.7) | 1 (2.4) |  | 4.5 (5.2) | 1.6 2.0) |  |
| 55-64 y | 11 (23.9) | 12 (28.6) |  | 20.9 (24.3) | 25.1 (31.9) |  |
| 65-74 y | 13 (28.3) | 16 (38.1) |  | 28.9 (33.6) | 25.9 (33.0) |  |
| 75-84 y | 15 (32.6) | 13 (31.0) |  | 28.7 (33.4) | 26.1 (33.2) |  |
| ≥85 y | 3 (6.5) | 0 (0.0) |  | 3.0 (3.5) | 0.0 (0.0) |  |
| Sex (%) |  |  | 0.001 |  |  | 0.679 |
| Male | 21 (45.7) | 34 (81.0) |  | 53.3 (62.0) | 53.1 (67.5) |  |
| Female | 25 (54.3) | 8 (19.0) |  | 32.7 (38.0) | 25.6 (32.5) |  |
| Race (%) |  |  | 0.140 |  |  | 0.755 |
| White | 28 (60.9) | 21 (50.0) |  | 54.5 (63.3) | 47.1 (59.8) |  |
| Black | 9 (19.6) | 16 (38.1) |  | 18.2 (21.2) | 22.1 (28.1) |  |
| Other | 9 (19.6) | 5 (11.9) |  | 13.3 (15.5) | 9.5 (12.1) |  |
| Marital status (%) |  |  | 0.703 |  |  | 0.920 |
| Married | 22 (47.8) | 23 (54.8) |  | 47.6 (55.3) | 40.4 (51.3) |  |
| Single | 7 (15.2) | 7 (16.7) |  | 11.8 (13.7) | 10.3 (13.1) |  |
| Other | 17 (37.0) | 12 (28.6) |  | 26.7 (31.0) | 28.0 (35.6) |  |
| Primary site (%) |  |  | 0.456 |  |  | 0.970 |
| Upper third of esophagus | 8 (17.4) | 5 (11.9) |  | 12.9 (15.0) | 9.7 (12.3) |  |
| Middle third of esophagus | 22 (47.8) | 18 (42.9) |  | 38.4 (44.6) | 37.1 (47.2) |  |
| Lower third of esophagus | 10 (21.7) | 8 (19.0) |  | 18.2 (21.2) | 14.5 (18.5) |  |
| Other | 6 (13.0) | 11 (26.2) |  | 16.5 (19.2) | 17.4 (22.1) |  |
| Tumor grade (%) |  |  | 0.618 |  |  | 0.989 |
| Grade I | 3 (6.5) | 5 (11.9) |  | 7.6 (8.8) | 7.4 (9.4) |  |
| Grade II | 31 (67.4) | 25 (59.5) |  | 55.0 (63.9) | 51.0 (64.7) |  |
| Grade III-Ⅳ | 12 (26.1) | 12 (28.6) |  | 23.5 (27.3) | 20.3 (25.8) |  |
| Tumor size (%) |  |  | 0.083 |  |  | 0.872 |
| ≤2 cm | 25 (54.3) | 14 (33.3) |  | 43.7 (50.9) | 35.5 (45.1) |  |
| >2 cm | 9 (19.6) | 8 (19.0) |  | 16.9 (19.7) | 15.7 (19.9) |  |
| Unknown | 12 (26.1) | 20 (47.6) |  | 25.4 (29.5) | 27.5 (35.0) |  |

Abbreviations: ESCC, esophageal squamous cell carcinoma; sIPTW, Stabilized inverse probability treatment weighting

| **Table S8. Baseline characteristics of endoscopic therapy and esophagectomy groups in EA before and after sIPTW** | | | | | | | | | | | | |  |
| --- | --- | --- | --- | --- | --- | --- | --- | --- | --- | --- | --- | --- | --- |
|  | | Unmatched | | |  | | After sIPTW | | | |  | |  |
| Group | | Endoscopic therapy | | Esophagectomy | *P* | | Endoscopic therapy | | Esophagectomy | | *P* | |  |
| N | | 148 | | 571 |  | | 700.9 | | 706.5 | |  | |  |
| Age (%) | |  | |  | <0.001 | |  | |  | | 0.977 | |  |
| <55 y | | 9 (6.1) | | 76 (13.3) |  | | 87.8 (12.5) | | 85.7 (12.1) | |  | |  |
| 55-64 y | | 20 (13.5) | | 182 (31.9) |  | | 182.1 (26.0) | | 202.7 (28.7) | |  | |  |
| 65-74 y | | 48 (32.4) | | 215 (37.7) |  | | 254.0 (36.2) | | 261.0 (36.9) | |  | |  |
| 75-84 y | | 57 (38.5) | | 94 (16.5) |  | | 159.0 (22.7) | | 141.8 (20.1) | |  | |  |
| ≥85 y | | 14 (9.5) | | 4 (0.7) |  | | 18.0 (2.6) | | 15.3 (2.2) | |  | |  |
| Sex (%) | |  | |  | 0.426 | |  | |  | | 0.329 | |  |
| Male | | 127 (85.8) | | 506 (88.6) |  | | 634.4 (90.5) | | 615.8 (87.2) | |  | |  |
| Female | | 21 (14.2) | | 65 (11.4) |  | | 66.5 ( 9.5) | | 90.7 (12.8) | |  | |  |
| Race (%) | |  | |  | 0.351 | |  | |  | | 0.131 | |  |
| White | | 144 (97.3) | | 543 (95.1) |  | | 688.5 (98.2) | | 675.9 (95.7) | |  | |  |
| Other | | 4 (2.7) | | 28 (4.9) |  | | 12.4 (1.8) | | 30.6 (4.3) | |  | |  |
| Marital status (%) | |  | |  | 0.067 | |  | |  | | 0.240 | |  |
| Married | | 89 (60.1) | | 400 (70.1) |  | | 421.8 (60.2) | | 484.8 (68.6) | |  | |  |
| Single | | 24 (16.2) | | 66 (11.6) |  | | 72.7 (10.4) | | 82.5 (11.7) | |  | |  |
| Other | | 35 (23.6) | | 105 (18.4) |  | | 206.4 (29.4) | | 139.3 (19.7) | |  | |  |
| Primary site (%) | |  | |  | 0.636 | |  | |  | | 0.632 | |  |
| Upper third of esophagus | | 2 (1.4) | | 4 (0.7) |  | | 6.6 (0.9) | | 6.1 (0.9) | |  | |  |
| Middle third of esophagus | | 10 (6.8) | | 27 (4.7) |  | | 26.9 (3.8) | | 32.0 (4.5) | |  | |  |
| Lower third of esophagus | | 120 (81.1) | | 481 (84.2) |  | | 620.3 (88.5) | | 597.3 (84.5) | |  | |  |
| Other | | 16 (10.8) | | 59 (10.3) |  | | 47.2 ( 6.7) | | 71.1 (10.1) | |  | |  |
| Tumor grade (%) | |  | |  | 0.211 | |  | |  | | 0.932 | |  |
| Grade I | | 23 (15.5) | | 81 (14.2) |  | | 89.6 (12.8) | | 100.7 (14.3) | |  | |  |
| Grade II | | 85 (57.4) | | 292 (51.1) |  | | 371.5 (53.0) | | 373.0 (52.8) | |  | |  |
| Grade III-Ⅳ | | 40 (27.0) | | 198 (34.7) |  | | 239.8 (34.2) | | 232.9 (33.0) | |  | |  |
| Tumor size (%) | |  | |  | <0.001 | |  | |  | | 0.675 | |  |
| ≤2 cm | | 92 (62.2) | | 302 (52.9) |  | | 418.0 (59.6) | | 389.6 (55.2) | |  | |  |
| >2 cm | | 17 (11.5) | | 207 (36.3) |  | | 185.6 (26.5) | | 223.0 (31.6) | |  | |  |
| Unknown | | 39 (26.4) | | 62 (10.9) |  | | 97.4 (13.9) | | 93.8 (13.3) | |  | |  |
| Adjuvant therapy (%) | |  | |  | <0.001 | |  | |  | | 0.487 | |  |
| Yes | | 30 (20.3) | | 12 (2.1) |  | | 39.6 (5.7) | | 30.3 (4.3) | |  | |  |
| No | | 118 (79.7) | | 559 (97.9) |  | | 661.3 (94.3) | | 676.2 (95.7) | |  | |  |
| Abbreviations: EA, esophageal adenocarcinoma; sIPTW, Stabilized inverse probability treatment weighting  **Table S9. Baseline characteristics of endoscopic therapy and chemoradiotherapy groups in EA before and after sIPTW** | | | | | | | | | | | | | |
|  | Unmatched | | | | |  | | After sIPTW | | | |  | |
| Group | Endoscopic therapy | | Chemoradiotherapy | | | *P* | | Endoscopic therapy | | Chemoradiotherapy | | *P* | |
| N | 148 | | 53 | | |  | | 204.2 | | 193.8 | |  | |
| Age (%) |  | |  | | | 0.869 | |  | |  | | 0.700 | |
| <55 y | 9 (6.1) | | 4 (7.5) | | |  | | 12.8 (6.3) | | 7.3 (3.7) | |  | |
| 55-64 y | 20 (13.5) | | 10 (18.9) | | |  | | 33.0 (16.2) | | 24.7 (12.7) | |  | |
| 65-74 y | 48 (32.4) | | 17 (32.1) | | |  | | 63.8 (31.2) | | 54.1 (27.9) | |  | |
| 75-84 y | 57 (38.5) | | 18 (34.0) | | |  | | 76.9 (37.6) | | 95.9 (49.5) | |  | |
| ≥85 y | 14 (9.5) | | 4 (7.5) | | |  | | 17.8 (8.7) | | 11.9 (6.1) | |  | |
| Sex (%) |  | |  | | | 0.518 | |  | |  | | 0.863 | |
| Male | 127 (85.8) | | 48 (90.6) | | |  | | 178.7 (87.5) | | 167.2 (86.2) | |  | |
| Female | 21 (14.2) | | 5 (9.4) | | |  | | 25.5 (12.5) | | 26.7 (13.8) | |  | |
| Race (%) |  | |  | | | 1.000 | |  | |  | | 0.652 | |
| White | 144 (97.3) | | 51 (96.2) | | |  | | 199.4 (97.6) | | 190.8 (98.4) | |  | |
| Other | 4 (2.7) | | 2 (3.8) | | |  | | 4.8 (2.4) | | 3.1 (1.6) | |  | |
| Marital status (%) |  | |  | | | 0.114 | |  | |  | | 0.136 | |
| Married | 89 (60.1) | | 33 (62.3) | | |  | | 126.2 (61.8) | | 142.2 (73.4) | |  | |
| Single | 24 (16.2) | | 3 (5.7) | | |  | | 25.9 (12.7) | | 7.2 (3.7) | |  | |
| Other | 35 (23.6) | | 17 (32.1) | | |  | | 52.0 (25.5) | | 44.4 (22.9) | |  | |
| Primary site (%) |  | |  | | | 0.154 | |  | |  | | 0.869 | |
| Upper third of esophagus | 2 (1.4) | | 4 (7.5) | | |  | | 9.1 (4.5) | | 7.1 (3.6) | |  | |
| Middle third of esophagus | 10 (6.8) | | 3 (5.7) | | |  | | 12.8 (6.3) | | 7.5 (3.9) | |  | |
| Lower third of esophagus | 120 (81.1) | | 40 (75.5) | | |  | | 161.7 (79.2) | | 163.7 (84.4) | |  | |
| Other | 16 (10.8) | | 6 (11.3) | | |  | | 20.6 (10.1) | | 15.6 (8.0) | |  | |
| Tumor grade (%) |  | |  | | | 0.041 | |  | |  | | 0.673 | |
| Grade I | 23 (15.5) | | 2 (3.8) | | |  | | 25.0 (12.2) | | 33.7 (17.4) | |  | |
| Grade II | 85 (57.4) | | 30 (56.6) | | |  | | 118.6 (58.1) | | 93.2 (48.1) | |  | |
| Grade III-Ⅳ | 40 (27.0) | | 21 (39.6) | | |  | | 60.6 (29.7) | | 67.0 (34.5) | |  | |
| Tumor size (%) |  | |  | | | <0.001 | |  | |  | | 0.945 | |
| ≤2 cm | 92 (62.2) | | 12 (22.6) | | |  | | 104.1 (51.0) | | 101.9 (52.6) | |  | |
| >2 cm | 17 (11.5) | | 21 (39.6) | | |  | | 39.5 (19.3) | | 39.2 (20.2) | |  | |
| Unknown | 39 (26.4) | | 20 (37.7) | | |  | | 60.7 (29.7) | | 52.8 (27.2) | |  | |

Abbreviations: EA, esophageal adenocarcinoma; sIPTW, Stabilized inverse probability treatment weighting
